# Influence of Thickness on the Magnetic and Magnetotransport Properties of Epitaxial La_0.7_Sr_0.3_MnO_3_ Films Deposited on STO (0 0 1)

**DOI:** 10.3390/nano11123389

**Published:** 2021-12-14

**Authors:** Simona Gabriela Greculeasa, Anda-Elena Stanciu, Aurel Leca, Andrei Kuncser, Luminita Hrib, Cristina Chirila, Iuliana Pasuk, Victor Kuncser

**Affiliations:** National Institute of Materials Physics, Atomistilor 405A, 077125 Magurele, Romania; simona.greculeasa@infim.ro (S.G.G.); anda.stanciu@infim.ro (A.-E.S.); aurel.leca@infim.ro (A.L.); andrei.kuncser@infim.ro (A.K.); luminita.hrib@infim.ro (L.H.); cristina.dragoi@infim.ro (C.C.); iuliana.pasuk@infim.ro (I.P.)

**Keywords:** thin films, Curie temperature, colossal magnetoresistance, magnetometry

## Abstract

Epitaxial La_0.7_Sr_0.3_MnO_3_ films with different thicknesses (9–90 nm) were deposited on SrTiO_3_ (0 0 1) substrates by pulsed laser deposition. The films have been investigated with respect to morpho-structural, magnetic, and magneto-transport properties, which have been proven to be thickness dependent. Magnetic contributions with different switching mechanisms were evidenced, depending on the perovskite film thickness. The Curie temperature increases with the film thickness. In addition, colossal magnetoresistance effects of up to 29% above room temperature were evidenced and discussed in respect to the magnetic behavior and film thickness.

## 1. Introduction

The discovery of ferromagnetism in polycrystalline perovskitic LaMnO_3_ structures [1] with a mixed valence of Mn ions (Mn^3+^/Mn^4+)^ has inspired numerous studies dedicated to La substituted perovskites. Among manganite perovskites, La_1−*x*_Sr_*x*_MnO_3_ attracts interest especially due to colossal magneto-resistance (CMR) effects [2,3]. La_1−*x*_Sr_*x*_MnO_3_ thin films are reported to present colossal magnetoresistance in high magnetic fields, e.g., 98% at 5 T, around 109 K [4] or ~99% at 7 T for temperatures < 120 K [5]. In addition, due to its high spin polarization, it is an interesting candidate to include in functional multilayers, such as multiferroic structures [6], in which epitaxy and thickness play a crucial role. Other effects include anisotropic magnetoresistance (AMR) [7], transport half-metallicity [8,9], the highest Curie temperature, *T_C_*, among the manganites (369 K for bulk [10]), metal-insulator transition [11], high magnetic anisotropy, low carrier density (10^21^–10^22^ cm^−3^) [12]. Moreover, the room temperature magnetocaloric effect (with different substitutions on the Mn site, such as Ni [13,14,15], Co [16] Cr, St, Ti [17] or with no substitutions at all [18,19]), large planar Hall effect in La_0.7_Sr_0.3_MnO_3_ films on MgO (1 0 0) [20], as well as magnetoelastic coupling at the interface between the La_2/3_Sr_1/3_MnO_3_ thin films and SrTiO_3_ (STO) substrate (due to the STO cubic-tetragonal transition [21]), can be noted. All of these above mentioned magneto-functionalities make La_1−*x*_Sr_*x*_MnO_3_ systems outstanding candidates for magnetic field sensors [22,23], high-density memory storage devices, tunnel magneto-resistance devices [24,25], pressure sensors [26], micro and nanomechanical resonant sensors [27], solid oxide fuel cells [28], spin injectors in organic spintronics [29] and references therein, non-volatile memory and memristive devices [30], spin light emitting diodes [31], organic light emitting devices (OLEDs) [32,33], spin wave resonance devices [34], uncooled infrared bolometers [35], microwave [36] and radio frequency [37] applications.

Tuning of the magnetic and transport properties can be achieved by controlling the La/Sr ratio in La_1−*x*_Sr_*x*_MnO_3_ [9,38]. The La ions are trivalent while depending on *x*, the Mn ions are showing a mixed valence behavior (Mn^3+^ and/or Mn^4+^). For *x <* 0.1, it is an antiferromagnetic (AFM) insulator, for 0.1 < *x* < 0.2, a ferromagnetic (FM) insulator and for 0.2 < *x* < 0.4, a FM metal. The coexistence of ferromagnetism and metallic conduction, as well as electron hopping between adjacent Mn^3+^ and Mn^4+^ along the Mn–O–Mn chains, are governed by the double exchange (DE) interaction [39,40] and electron-phonon coupling [9]. In addition, a sharp drop in resistivity occurs along with the paramagnetic-to-ferromagnetic transition for 0.2 < *x* < 0.4 [41]. In the concentration range of interest for the present study (*x* = 0.3), the following features should be noticed: The Mn-O-Mn bond angle is 166.3° for *x* = 1/3 and an average Sr-site radius of 1.24 Å [42], the conduction band is more than half-filled and the spin polarization at the Fermi level is 100% [43,44].

Magnetic properties, including *T_C_* are influenced by the sample geometry: *T_C_* values of 369 K [10] for La_1−*x*_Sr_*x*_MnO_3_ single crystals (0 ≤ *x* ≤ 0.6) and 451 K for La_0.67_Sr_0.33_MnO_3_ epitaxial films on MgO [45] were obtained. In thin film geometry, the film thickness also plays an important role in influencing the Curie temperature. An increase of *T_C_* with thickness for La_0.7_Sr_0.3_MnO_3_ films on STO (0 0 1) was reported for thicknesses in the range of 3–40 unit cells (1.2–16 nm; cell parameter ~0.4 nm), from 80 K (1.2 nm) to 320 K (16 nm) [46] and for La_0.8_Sr_0.2_MnO_3_ films on LSAT between 250 and 350 K for thicknesses between 7 and 43 nm [47]. A severe reduction of *T_C_* with thickness [48] or non-monotonic variations [49] can be related to strain, stress relaxation, and defects. The substrate mismatch can also induce variations of *T_C_* or *T_P_* (metal-insulation transition temperature). Another study reported differences between *T_C_* values of La_0.67_Sr_0.33_MnO_3_ films deposited by PLD on various substrates, such as GSO, STO, and NGO: 328, 334, and 338 K [50]. *T_P_* was found to increase with thickness, increase with compressive strain, and decrease with tensile strain [51]. When deposited on (Pb_0.97_La_0.02_) (Zr_0.58_Sn_0.3025_Ti_0.1175_)O_3_ (PLZST) ceramics, the La_0.7_Sr_0.3_MnO_3_ films have *T_C_* values increasing with thickness reduction [52]. The presence of oxygen vacancies, as well as strain induced by a STO layer were found to increase considerably the *T_C_* of La_0.7_Sr_0.3_MnO_3_ film deposited on a STO substrate [53], which also eliminates magnetic dead layers [54].

Magnetoresistance (MR) effects in doped manganites have been analyzed in terms of super-imposed (multiple) mechanisms, such as the DE interaction [55], electron-phonon coupling which explains polaron effects in transport studies [56] or orbital ordering effects [57]. Moreover, the existence of phases with different electronic densities and percolative processes contribute significantly to the MR phenomena. In particular, in the vicinity of the metal-insulator transition [58]. A phase separation was observed by scanning tunneling spectroscopic studies of La_1−*x*_Ca_*x*_MnO_3_ (with *x* ≈ 0.3) proving a dominant insulating behavior above the bulk transition temperature (*T_C_*) and mixed metallic (ferromagnetic) and insulating (paramagnetic) phases below *T_C_* [59]. First investigations on the transport properties of manganese perovskites explained their complex phase diagram and the metal—insulator transition on the basis of the DE interaction that favors a change in resistivity due to electron scattering effects, which are enhanced in the high temperature phase (paramagnetic phase) and are diminished in the low temperature phase (ferromagnetic phase) [43]. It has been concluded that the mixed-phase tendencies originate from: (i) Electronic phase separation between phases with different densities involving the formation of nanometer scale coexisting clusters, and (ii) disorder-induced phase separation with percolative characteristics between equal-density phases in the vicinity of the metal-insulator transition that leads to the formation of micrometer sized clusters [9]. In this respect, the transition from the metallic state to the isolator one is progressive, taking place over a large temperature interval and cautions in comparing with the magnetic transition temperature should be considered.

In this article, changes in magnetic and magneto-transport properties of epitaxial La_0.7_Sr_0.3_MnO_3_ (LSMO) films in relation to film thickness, are analyzed. The films were deposited on SrTiO_3_ (STO) substrates by pulsed laser deposition (PLD) and were characterized by X-ray diffraction (XRD) and transmission electron microscopy (TEM) with respect to morpho-structural aspects. The magnetic characterization has been performed by superconducting quantum interference device (SQUID) magnetometry, whereas the magneto-conduction experiments have been performed by a physical property measurement system (PPMS). Special attention was given to the correlation of the magnetic and magneto-resistance behavior in terms of the thickness dependent progressive metal-insulator transitions observed in the investigated films. The purpose and novelty of this study are related to the large field of applications of epitaxial LSMO thin films, including functional multilayers in which epitaxy and thickness play a crucial role. These parameters influence the electronic structure and numerous related effects and are also reflected in the magnetic and magneto-conduction behavior, directly or through connected phase transitions. The advantage of the considered LSMO system is its simplicity, as well as the reliability in controlling the targeted parameters and in subsequent theoretical modelling.

## 2. Materials and Methods

La_0.7_Sr_0.3_MnO_3_ samples with different thicknesses, *t*, ranging from 9 to 90 nm, were deposited on STO (0 0 1) substrates by the pulsed laser deposition method (PLD) using a KrF laser (λ = 248 nm). Inside the deposition chamber, the substrate temperature was maintained at 800 °C and the oxygen pressure at 0.27 mbar. The LSMO target from Praxair was ablated with a frequency of 1 Hz and a fluence of 2 J/cm^2^. After deposition, the film was cooled to room temperature in high oxygen pressure with a slow ramp. The samples will be denoted according to their thickness: LSMO 9, 30, 36, 60, and 90, respectively. Excepting the thickness of sample LSMO 90, the thicknesses of all the other samples have been determined by TEM. The obtained thicknesses nicely matched with thicknesses expected from previous calibration with respect to the number of pulses in identical deposition conditions. Moreover, in the case of samples LSMO 9 and LSMO 36, the TEM results perfectly agree with thicknesses obtained by XRD. The thickness of the thickest sample was estimated only by calibration with the number of pulses.

The XRD measurements were performed with a Bruker D8 Advance diffractometer with Cu Kα radiation in medium resolution parallel beam setting.

Cross-sectional images were acquired using a JEM ARM-200F analytical electron microscope, equipped with a spherical aberration corrector for the scanning electron transmission microscopy (STEM) mode. Within the dark field STEM mode (STEM-DF), the images are due to electrons, which are inelastically scattered by the sample, as detected via an annular dark field detector. The STEM-DF images show a contrast proportional to the square of the local atomic number. Consequently, whiter areas are related to a higher local Z number, whereas the darker areas are related to a lower local Z number.

Magnetic hysteresis loops collected in parallel and perpendicular geometry (field applied in the film plane and perpendicular to the film plane, respectively) and temperature dependent magnetization data were obtained with a MPMS SQUID magnetometer (Quantum Design) working at temperatures as low as 2 K and in applied fields of up to 7 T. The current experiments were conducted in the temperature range of 10–340 K and in applied fields of 1 T, using the most sensitive option (RSO).

The four point collinear probe method [60] was implemented on a PPMS (Quantum Design) in order to measure magnetoresistance (MR) curves at various temperatures, as well as the temperature dependence of the resistivity. Excitation currents of 10–20 μA were passed through the two external probes, whereas the voltage drop was collected through the two internal probes (in line contacts) separated by 5 mm. The MR measurements were performed in parallel and perpendicular geometry.

## 3. Results and Discussion

**Figure 1:**
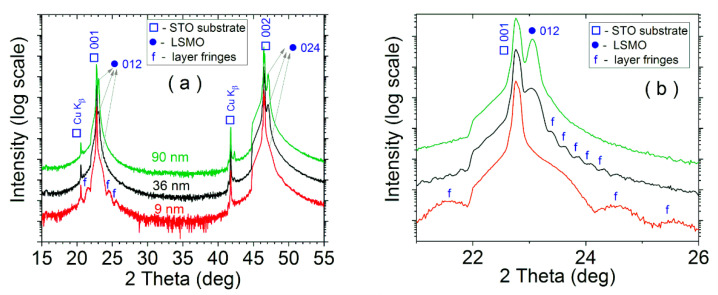
X-ray diffraction patterns of the LSMO samples: 9, 36, and 90 nm (**a**). Enlarged view of the layer fringes (**b**).

The XRD patterns of samples LSMO 9, 36, and 90 are shown in Figure 1. The rhombohedral structure of LSMO, belonging to the R3c:H space group [61], and the cubic structure of STO, corresponding to the Pm3m space group [62], are evidenced. The LSMO lines are clearly evidenced for the two thicker films, while for the 9 nm film, the Bragg peaks are hardly visible since the profiles are too large and superimposed on the substrate ones. The films show a complete texturing with the (012) planes parallel to the substrate. In the case of the thinner films, the layer fringes (Pendellösung fringes) are an indirect evidence of LSMO epitaxial layers, with smooth interfaces and well-ordered lattice over the whole film. The layer thicknesses determined from the period of the fringes are 9 and 36 nm. No fringes were observed for the thickest sample.

**Figure 2:**
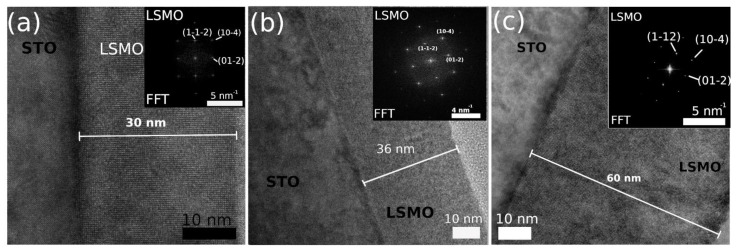
Cross-sectional STEM-DF micrographs of LSMO 30 (**a**), LSMO 36 (**b**), and LSMO 60 (**c**) films grown on the STO substrate. The corresponding Fourier Transform patterns belonging to the LSMO structures are shown in the right side insets of each figure.

The microstructure of the samples LSMO 30, 36, and 60 is evidenced in the cross-sectional STEM-DF micrographs and selected area (electron) diffraction (SAED) patterns presented in Figure 2. The epitaxial character of the La_0.7_Sr_0.3_MnO_3_ films (especially for reduced thicknesses), as well as the flat film-substrate interface are revealed. The corresponding SAED patterns shown in the inset indicate the formation of LSMO as an unique phase of the film on the STO substrate. The Miller indices of three main diffraction rings are indicated on the SAED pattern. The diffraction spots are arranged in a square lattice, suggesting the high crystalline quality of the films.

**Figure 3:**
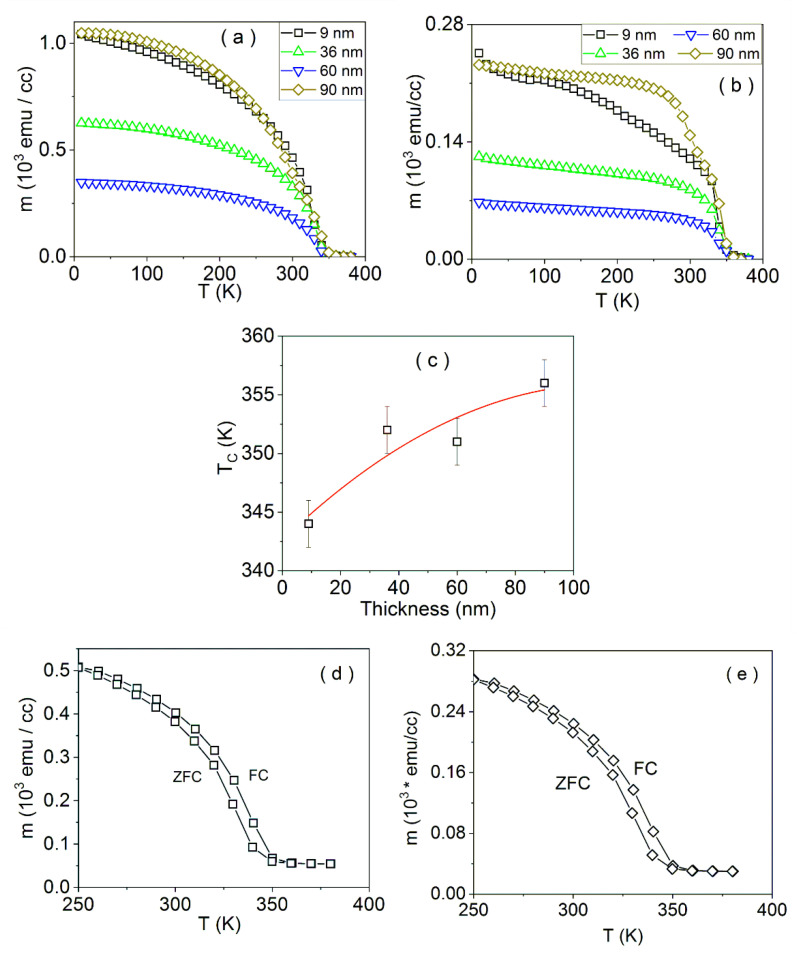
The temperature dependence of the magnetic moment per unit volume, in parallel (**a**) and perpendicular (**b**) geometry, in a cooling field (*H_FC_*) of 1 kOe, for the LSMO samples. Curie temperature for the LSMO samples (**c**); the line connecting the experimental points is only for guidance. ZFC-FC curves of samples LSMO 36 (**d**) and LSMO 60 (**e**), with field cooling at 500 Oe, in the parallel geometry.

The temperature dependence of the magnetization per unit volume (cube centimeter(cc)) for LSMO samples of different thicknesses, in parallel and perpendicular geometry, in a cooling field (*H_FC_*) of 1 kOe, is shown in Figure 3a,b, respectively. The magnetic contribution of the STO substrate, probably related to impurity contributions and a small defect-related temperature independent component [63], was not taken into consideration due to the negligible signal (a few orders of magnitude lower). At decreasing temperature, the magnetic moment starts to increase, indicating the transition to the ferromagnetic state of the LSMO oxide. A very important aspect is that the magnetic moment in the parallel geometry is a few times higher than in the perpendicular geometry, due to the fact that the applied field of 1 kOe is sufficient to rotate all the magnetic spins in the film plane (parallel geometry), but not enough to rotate all of the spins in the out-of-plane (perpendicular) geometry, as a first experimental evidence of the in-plane anisotropy of the films. More striking is the variation of the magnetization at low temperatures with the film thickness. It decreases substantially with the film thickness up to 60 nm and then increases consistently for the thickest film of 90 nm. As a possible explanation is the reconfiguration of the electronic structure of the films with the thickness dependent average strain induced by the STO substrate. The poorer epitaxy of the thickest film may reverse the initial tendency. Specific further electron structure calculations are necessary in order to support this hypothesis.

The transition temperature vs. the film thickness, presented in Figure 3c as obtained from the temperature dependence of the magnetic moment per unit volume (tangential method, average of the values corresponding to the parallel and perpendicular geometries), shows an overall increase with the film thickness, already reported in [46]. However, with a saturation tendency for thicknesses above 60 nm. The values are typical for LSMO thin films and lower than the bulk-like transition temperature of 369 K [10].

In Figure 3d,e, the ZFC-FC curves of sample LSMO 36, in the parallel geometry, are shown. The sample was cooled in zero field from room temperature to 250 K. The ZFC curve was registered while heating the sample from 250 to 380 K in 500 Oe applied field, whereas the FC curve was registered while cooling the sample from 380 to 250 K in the same applied field. For the samples LSMO 36 and 60, the FC curve is above the ZFC curve, indicating that the applied 500 Oe field is sufficient to exclude the domain structure, suggesting low in-plane anisotropies. A considerable decrease of the magnetic moment is observed in both curves by approaching 350 K, supporting the LSMO transition towards the paramagnetic state, as also evidenced in Figure 3a,b.

**Figure 4:**
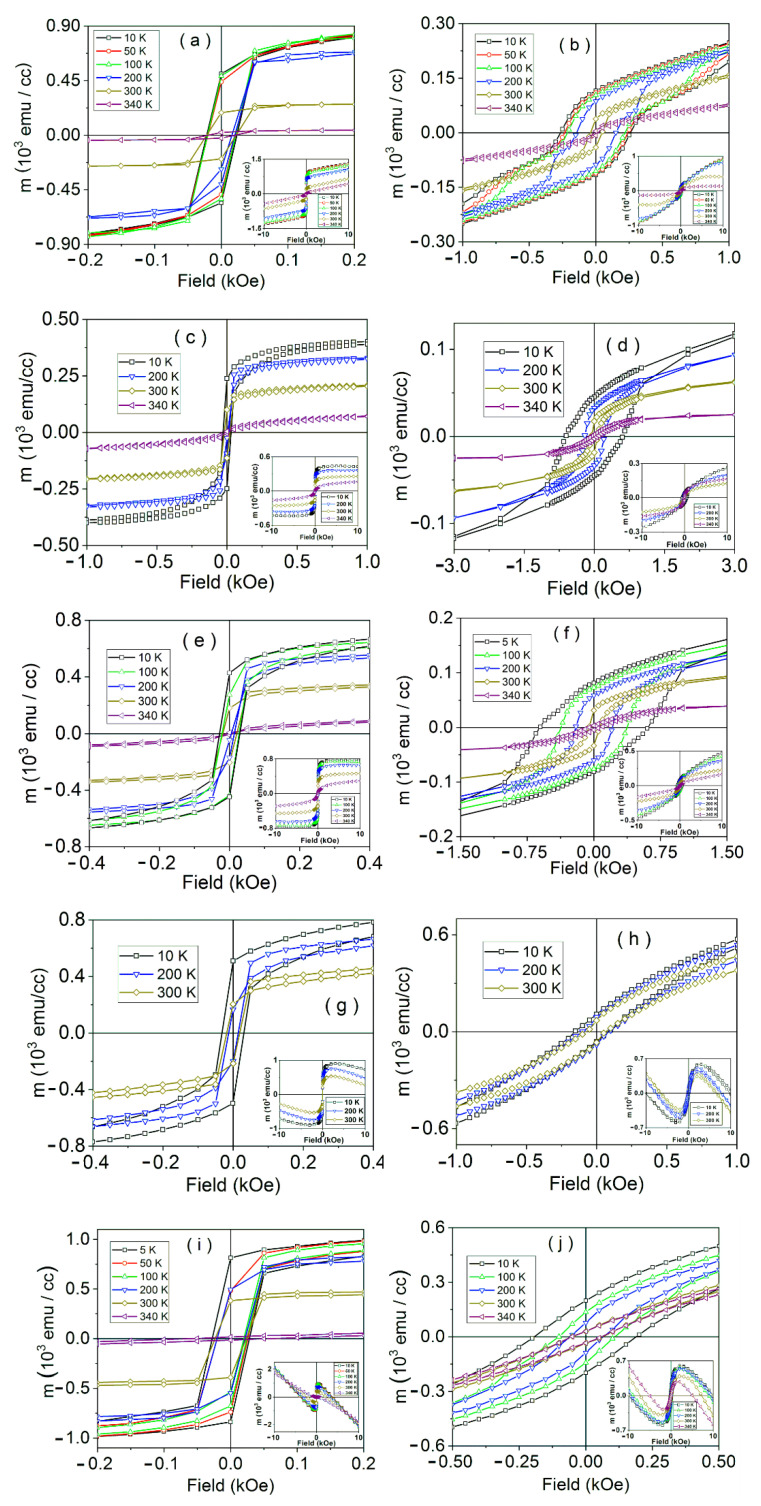
Magnetic hysteresis curves in the low field range of samples: LSMO 90 in parallel (**a**) and perpendicular (**b**) geometry; LSMO 60 in parallel (**c**) and perpendicular (**d**) geometry; LSMO 36 in parallel (**e**) and perpendicular (**f**) geometry; LSMO 30 in parallel (**g**) and perpendicular (**h**) geometry; LSMO 9 in parallel (**i**) and perpendicular (**j**) geometry. The corresponding raw curves in the high field range are shown as insets. The linear diamagnetic component due to the substrate was extracted in the main graphs.

The magnetic hysteresis curves of samples LSMO 90, 60, 36, 30, and 9, in parallel and perpendicular geometry, are shown in Figure 4. The linear component was extracted from the raw hysteresis curves shown in the corresponding insets. Two spin structures of different in-plane and out-of-plane anisotropies are evidenced in the hysteresis curves of the LSMO 90 sample, which may be assigned to an interface component and a main film component. These magnetic heterogeneities inside thin magnetic films may be similar to the results obtained by [64]. The first component is characterized by a higher coercive field (interface component), while the second component presents a softer magnetic behavior (main component). The interface spins are harder to rotate, suggesting a disordered spin structure, relative to the main film. These features are more clearly observed in the perpendicular geometry (see inset of Figure 4b), where spins cannot be saturated at lower temperatures. In the case of the samples LSMO 60 and 36, different in-plane and out-of-plane rotations are observed, as well for the two magnetic contributions, with interface spins harder to rotate, similar to the LSMO 90 sample.

For samples LSMO 9 and 30, two different magnetic contributions are clearly evidenced in the parallel geometry (see Figure 4g,i). For LSMO 9, the first magnetic phase, characterized by a higher coercive field, is assigned to the interface component, and is found in a proportion of about 25%, while the second component, with a contribution of approximately 75%, presents a softer magnetic behavior. Similar to the case of the thicker samples, in-plane anisotropies of the two phases are very different: The spins corresponding to the main film are more easily rotated than the interface spins. Considering the hysteresis curves in the perpendicular geometry, the two magnetic phases are no longer distinctly observed, suggesting that the out-of-plane anisotropies are similar. It is worth mentioning that in sample LSMO 36 the interface phase, as calculated from the hysteresis loop in parallel geometry is about 20%, whereas in LSMO 90, it is almost indistinguishable, as expected from a significant interface/volume ratio.

**Figure 5:**
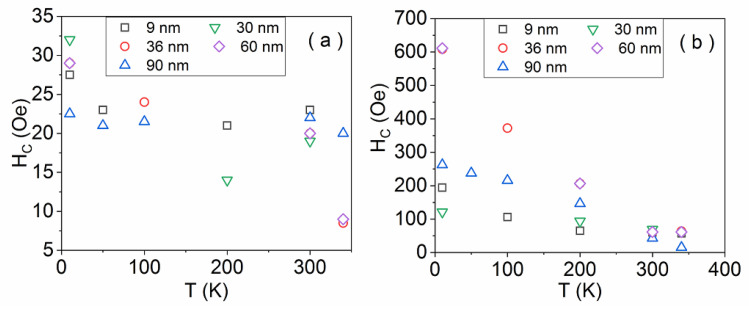
Temperature dependence of the coercive field of the main magnetic phase in parallel (**a**) and perpendicular (**b**) geometry for the LSMO samples.

The temperature dependence of the coercive field for the main component of the film in parallel and perpendicular geometry, for the LSMO samples, is shown in Figure 5a,b, respectively. In the parallel geometry, the coercive field values are considerably reduced (few tens of Oe) in relation to the values in the perpendicular geometry (more than 600 Oe).

In the frame of the double exchange interaction, there is an electron transport process between Mn^3+^ and Mn^4+^ ions mediated by O^2−^ ions in LSMO films. In principle, below the metal-insulator transition temperature, *T_P_*, the charge, spin, and magnetic ordering are governed by the electron–phonon coupling interactions and the coulomb repulsion between electrons, resulting in the FM behavior with low resistivity. As the temperature increases towards *T_P_*, the thermal motion starts to affect the magnetic ordering, leading towards the PM behavior and weakening of the electron transport. This should result in an increase of resistivity specific to a metallic state before *T_P_* and a sharp increase of the resistivity above *T_P_*, namely in the insulating state. Nevertheless, that would be true in the case of a sharp metal-insulator transition, superposing over the magnetic-paramagnetic transition.

**Figure 6:**
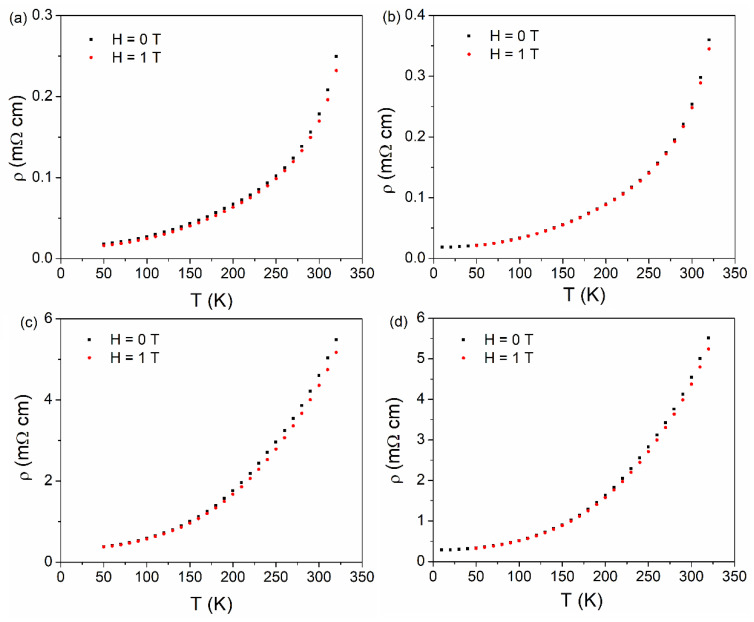
Resistivity as a function of temperature of the samples: LSMO 30 (**a**,**b**) and LSMO 60 (**c**,**d**) collected in parallel (**a**,**c**) and perpendicular (**b**,**d**) geometry.

The variation of resistivity with temperature in the temperature range of 10–320 K, in parallel and perpendicular geometry, for different film thicknesses (30 and 60 nm, respectively), is shown in Figure 6. Of note, the slow and almost linear increase of the resistivity at low temperatures is followed by a much faster increase at high temperature. No exponential decrease of the resistivity with temperature, specific to band gap semiconducting/insulating compounds are observed. These peculiarities of the temperature dependence of resistivity suggest, on the one hand, a progressive transition to an insulating state and, on the other hand, a not complete transition to the insulating state under the maximum measuring temperature. The resistivity increases steeply as the metal insulator transition is approached due to an increase of the spatial extension of the paramagnetic insulating regions. These assumptions can be correlated to the thermomagnetic curves (Figure 3) where a decrease of the magnetization in the temperature interval corresponding to the changing behavior in ρ (T) curves is observed. Of note, there are difficulties in defining *T_P_* in the conditions of this progressive transition, as well as in the way to relate it to *T_C_*. Assuming that *T_P_* is the temperature where the transition to the insulating state is complete, it would be experimentally defined by the temperature of maximum resistivity (above *T_P_* the resistivity will decrease). A maximum in the ρ (T) curves was observed in [65] where specific conduction mechanisms belonging to different temperature intervals were also proposed. Although in the present case the metal-insulator transition is not complete, qualitative specific behaviors can be noted from Figure 6. Accordingly, a lower resistivity of the metallic state and a much sharper transition is observed for the LSMO 30 film as compared to the LSMO 60 film. On the other hand, it is observed that ρ (T) curves depend also on the applied magnetic field. The differences between the resistivity in 0 and 1 T applied field is evident in a large amount at higher temperatures (approaching the metal-insulator transition).

The magnetoresistance, *MR*, quantifies the effect of an external magnetic field on the electrical behavior of the samples and will be defined in the following by: *MR*(*H*) *=* [*R*(*H*) *− R*(*H_max_*)]/*R*(*H_max_*) *×* 100%, where *R*(*H*) is the resistance in a magnetic field *H* and *R*(*H_max_*) is the resistance at the maximum applied field *H_max_*.

**Figure 7:**
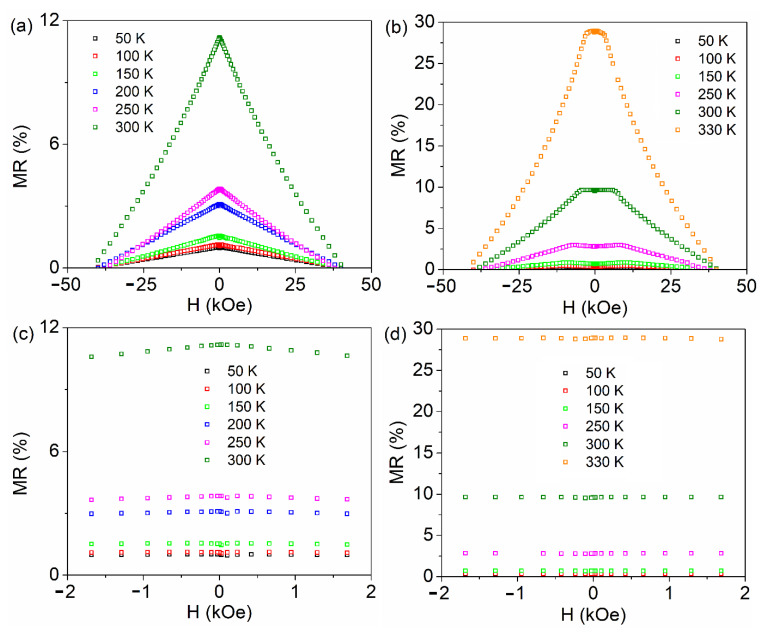
MR curves of LSMO 30 sample collected in parallel (**a**) and perpendicular (**b**) geometry; MR curves collected in parallel (**c**) and perpendicular (**d**) geometry in the low field region.

**Figure 8:**
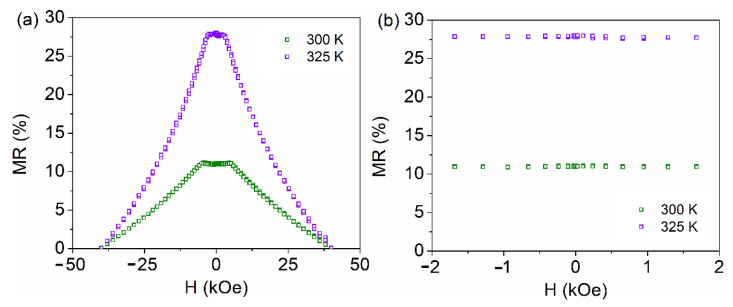
MR curves of LSMO 36 sample collected in perpendicular geometry (**a**). MR curves collected in perpendicular geometry in the low field region (**b**).

**Figure 9:**
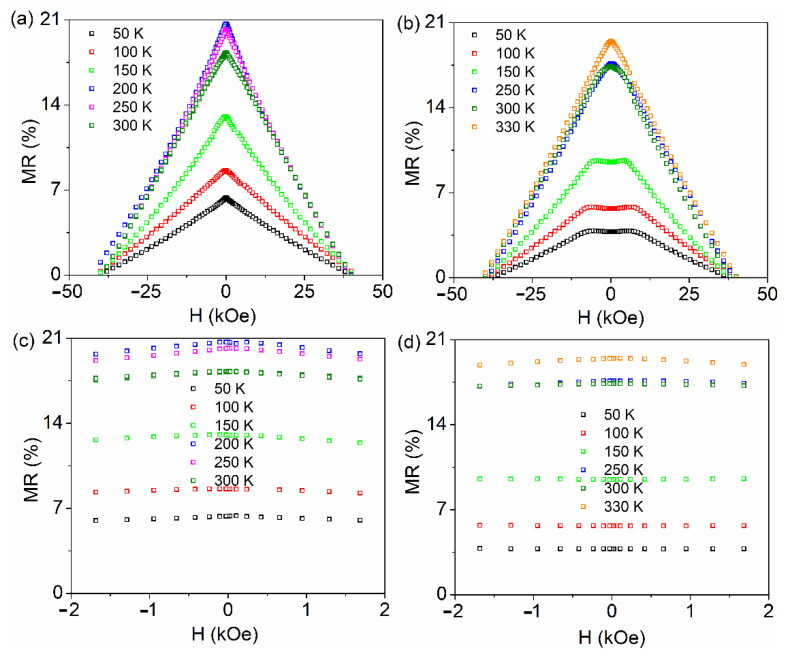
MR curves of LSMO 60 sample collected in parallel (**a**) and perpendicular (**b**) geometry. MR curves collected in parallel (**c**) and perpendicular (**d**) geometry in the low field region.

**Figure 10:**
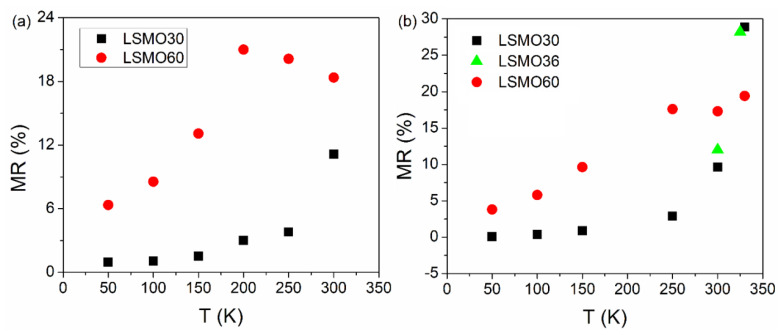
MR effect at different temperatures in parallel (**a**) and perpendicular (**b**) geometry for LSMO 30 and LSMO 60.

The magnetoresistance of samples LSMO 30, 36, and 60 with the applied field in the parallel and perpendicular geometry, calculated by the above relation is shown in Figure 7, Figure 8 and Figure 9, respectively. The typical colossal magnetoresistance (CMR) effects in manganites are clearly observed, with specific features depending on geometry as a result of the different magnetic reversal processes. The magnetoresistance effect is more pronounced in the perpendicular geometry and at high temperatures for sample LSMO 30, while it decreases for higher film thickness (e.g., in the case of sample LSMO 60). The MR effect as a function of temperature for LSMO 30, 36, and 60 measured in parallel and perpendicular geometry is represented in Figure 10. The MR effect is enhanced at 300 K due to the electron scattering on paramagnetic regions that become dominant when approaching room temperature.

MR curves of LSMO 30, 36, and 60 samples in perpendicular geometry present a plateau near the 0 field region at temperatures in (50–330 K), (300–325 K), and, respectively, (50–150 K) intervals, at variance to the case of parallel geometry, where only a maximum at 0 T applied field is observed, more pronounced at higher temperatures. This temperature dependent feature was previously associated (and supported by electron microscopy data) to the prevalence of insulating regions and of ferromagnetic domains in the corresponding temperature range [66]. As a general observation, the MR effect increases sharply at higher temperatures, approaching *T_C_*. This behavior is more evident in the case of the thinner films with a sharper metal-insulator transition.

## 4. Conclusions

In this study, the influence of thickness on the magnetic and magneto-transport properties of La_0.7_Sr_0.3_MnO_3_ films is investigated. The LSMO films were prepared by PLD and analyzed by XRD, SQUID magnetometry, and PPMS. Two magnetic components are evidenced in the hysteresis loops of the LSMO films, one, of higher coercivity which is assigned to the closest interface with the substrate and the second one, which is dominant, to the rest of the film. The relative contribution of the interfacial component modifies the overall magnetic properties of the films with respect to both anisotropy and magnetization aspects. *T_C_* increases with the film thickness with a saturation tendency at about 355(3) K for thicknesses above 60 nm. Temperature dependent resistivity measurements indicate a progressive metal-insulator transition. The progressive extension of the insulating regions at higher temperatures impose a much sharper increase of the resistivity with respect to the linear increase specific to the metallic state at low temperature. However, the achieved temperatures during the magneto-conduction experiments were not high enough for a complete transition to the insulating state. The transition is sharper in thinner films, of higher relative contribution of the interfacial component. The electrical transition is correlated to the magnetic one. Typical colossal magnetoresistance effects, in which the magnitude and shape depend on the measuring geometry as a result of the different magnetic reversal processes, were observed. The MR effect increases with temperature. The highest values are obtained at temperatures closer to the metal-insulator transition superposed over the magnetic one. A sharper increase of the MR effect above 300 K is observed in the thinner films, whereas a smoother increase but with higher values at 300 K is observed for the thicker films. Typical values for the CMR effect at 300 K are: 11% and 18% for LSMO 30 in parallel and perpendicular geometry, respectively and 9% and 17% for LSMO 60 in parallel and perpendicular geometry, respectively. The maximum CMR effect of 29% was obtained at the highest measuring temperature of 325 K for the LSMO 30 sample, in perpendicular geometry. The most triangular shape, of higher interest in applications, is usually obtained in parallel geometry, as well as in perpendicular geometry close to the paramagnetic transition, where the coercive field becomes negligible.
